# Psychosocial and Physiological Health Outcomes of Green Exercise in Children and Adolescents—A Systematic Review

**DOI:** 10.3390/ijerph16214266

**Published:** 2019-11-02

**Authors:** Carina Mnich, Susanne Weyland, Darko Jekauc, Jasper Schipperijn

**Affiliations:** 1 Institute of Sports and Sports Science, Karlsruhe Institute of Technology, 76149 Karlsruhe, Germany; susanne.weyland@kit.edu (S.W.);; 2 Department of Sport Science and Clinical Biomechanics, University of Southern Denmark, 5230 Odense, Denmark

**Keywords:** green exercise, physical activity, nature, children, adolescents, outdoors

## Abstract

Both physical activity (PA) and nature exposure are associated with several youth health benefits. However, the health outcomes when being physically active in nature, called Green Exercise (GE), are less clear. Thus, the purpose of this systematic review was to provide an overview of the psychosocial and physiological outcomes of GE in children and adolescents and to outline future GE research directions. The PRISMA statement guided the review. Web of Science, PubMed, ERIC, and APA PsychNET were systematically searched in February 2019, including studies between 2000 and 2019. Fourteen of 1175 identified publications were included, which reported 15 different psychosocial and six different physiological outcomes, with some studies reporting more than one outcome. For 16 outcomes, studies reported either similar or no effects for both GE and comparison groups. For six outcomes, studies reported stronger effects for GE, for three outcomes, studies reported stronger effects in the comparison group. Evidence was rated as weak, using the EPHPP tool. Thus, GE does not have deleterious effects for children and adolescents compared to PA in other settings. GE might be beneficial; however, due to the study’s heterogeneity and quality, it is premature to make definite conclusions. Future research should build the quality of evidence for GE, use more rigorous research designs, and investigate the underlying effects and mechanisms of GE.

## 1. Introduction

Physical activity (PA) is associated with numerous health benefits in children and adolescents, including improved cardiovascular health, mental health, bone strength, fitness levels, weight, and quality of life [1,2]. PA also impacts children’s cognitions, resulting in improved achievements at school [3] and improved cognitive functions [4]. In addition, PA during youth is related to long-term benefits in adulthood, including a reduced risk of depression, hypertension, and type 2 diabetes [5,6], making PA a core aspect of youths’ short- and long-term health.

Natural environments are also associated with positive effects for youth. Access to green spaces is associated with improved mental and general well-being and lower stress [7,8], lower depression rates in children [9], milder symptoms of ADHD [10] as well as improved cognitive and emotional outcomes [11,12,13]. Green spaces are also related to fewer behavioral problems [14], hyperactivity, and peer and conduct disorder problems [15,16]. Looking at physical health outcomes, green spaces are associated with longer sleep [8], lower blood-pressure [17,18], and lower rates of overweight, obesity, and sedentary behavior [19] in children.

A body of research has already explored the role of the natural environment for children’s PA. One study showed that children between 8–14 years who experience more than 20 min of daily exposure to green spaces engaged in nearly five times more daily MVPA than children without daily exposure to green spaces [20]. Four other studies revealed that outdoor time in children aged 3–14 years has positive effects on PA, sedentary behavior, and cardiorespiratory fitness [21,22,23,24]. However, the causality of the found relations is unclear due to a lack of RCTs.

Having shown the benefits of both natural environments and PA individually, there might be sub-additive, additive, or synergistic effects of combining both components [25]. Green exercise (GE) is defined by Pretty and colleagues as “adopting physical activities whilst at the same time being directly exposed to nature” [26]. Accordingly, GE does not only comprise PA taking place in green environments (e.g., parks and forests), but also in blue spaces (e.g., rivers and lakes) and any other environment containing natural components. Pretty also distinguished between different levels of engagement with nature: viewing nature (e.g., looking at a forest picture), being in the presence of nature incidentally while engaged in other activities (e.g., cycling to school), and involvement and participation in nature (e.g., trail running), with all three levels shown to impact mental health [27]. Regarding behavior, GE includes both PA as well as planned and structured exercise in green settings.

The benefits of GE have already been explored in adults. A systematic review by Lahart and colleagues compared the effects of indoor and outdoor PA for physical and mental-wellbeing in adults. Results indicated lower perceived exertion scores for GE and a better response for affective valence. Findings about other outcomes were inconsistent [28]. Other studies have shown better restorative effects and affective responses [29], improved mental health [30], and reduced state anxiety [31,32] for GE compared to exercise indoors or in concrete environments. While one could argue that these effects appear after any exercise program, another study found that GE had a greater influence on improved mood and stress scores than exercise alone [33].

As these studies only included adult participants, less is known about how GE impacts children’s physiological and psychosocial health outcomes. Various studies have examined the relationship between the natural environment and PA, but only few studies have looked at the benefits beyond increased PA levels. Four systematic reviews were found that investigated benefits of physical activity in natural settings, but they mostly include adults and if they also included children and adolescents, they did not treat them as a separate target group [28,34,35,36].

The aim of this work is to fill this research gap. Therefore, this systematic review serves three purposes: (1) Provide an overview about the physiological and psychosocial outcomes of PA in natural environments (GE) in children and adolescents. (2) Demonstrate the effectiveness of GE in terms of the outcomes assessed. (3) Based on the overview of existing evidence, outline future research directions to study GE in children and adolescents.

## 2. Materials and Methods

The PRISMA Statement has been used for this systematic review [37] and the study protocol has been registered with PROSPERO (CRD42019136385).

### 2.1. Search Strategy and Eligibility Criteria

A systematic literature search was conducted on 11th February 2019, using the databases Web of Science (All Databases), PubMed, APA PsychNET, and ERIC. The primary search was based on title, abstract and keywords, using Boolean logic for the combination of search terms. Additional, possibly relevant studies were identified using the “snowball principle” by screening the references of all included studies [38] and of the four systematic reviews that had already been carried out in this field [28,34,35,36].

Search terms were based on previous reviews and agreement between the first and third author, resulting in a search strategy with three parts with synonyms for (1) nature, (2) PA and exercise, and (3) children and adolescents. The search has not been restricted to certain outcomes to allow for the inclusion of a comprehensive body of literature. Search strategies for all databases can be found in the study protocol; as an example, the following strategy had been used in the Web of Science database: “Title = (green OR natur* OR outdoor OR outside OR park OR green space*) AND Title = (physical* active* OR exercise* OR walk* OR cycl* OR hik* OR leisure time OR leisure-time OR recreation*) AND Topic = (child* OR adolescen* OR youth OR young people OR student* OR pupil*)”.

The components of the PICOS question, including the components population, intervention, comparison, outcomes, and study design, were answered to define the eligibility criteria and are presented in Table 1. Beyond the PICOS question, only single-study articles published in peer-reviewed journals in English language between 2000 and 2019 were considered. This time period was chosen due to the fact the definition of GE was published in 2003 [26]. Considering the conceptual development and the publication process that it takes until a manuscript is published, such as the publication with the GE definition, three more years before the actual publication were included.

### 2.2. Screening and Study Selection

Reference results of the database search were exported to the reference program EndNote and duplicates removed. Studies were screened for inclusion criteria based on title in a first step, followed by abstract and full-text screening. The screening process was conducted independently by the first two authors. The two authors discussed their results, and full-texts were included in the analysis based on mutual agreement. References of the included studies were scanned for other relevant articles independently, the results discussed, and studies included based on the first two authors’ mutual agreement. If there was no consent, a third author was consulted for a final decision. Relevant data about the included articles was extracted by one author, comprising authors and year, study design, country of study and participants, type of GE and procedure, outcomes, outcome measurements, and results including the main quantitative results. The second author then reviewed the data extraction sheets. Included studies were sent to a member of the “Green exercise research group” of the University of Essex (UK) who gave feedback about any other studies familiar to him in this area.

The “Effective Public Health Practice Project” (EPHPP) was used for bias risk assessment of the included studies [42]. The tool was applied to the included studies independently by two authors and the final rating determined based on consensus. The EPHPP tool can be used for observational, cross-sectional, pre-post, cohort, and randomized controlled trial designs [43] and has, for example, been used previously in a systematic review assessing health outcomes of e-bike use [44]. The EPHPP tool has six equally weighted categories that are included in an overall-rating to assess the study quality: selection bias, study design, confounders, blinding, data collection methods, and withdrawals and dropouts. The withdrawal and dropout-category was also applied to cross-sectional studies as this contains information about the percentage of participants that completed the study. Data collection methods were considered as reliable and valid if at least 50% of the measurement instruments used in the study were reported as valid and reliable.

Each category received a strong (1), moderate (2), or weak rating (3), which was the basis for the overall rating of the study: strong (no weak ratings), moderate (one weak rating), and weak (two or more weak ratings). Two additional categories, intervention integrity and analyses, are included in the tool, but not in the overall rating [42]. Statistical methods were reported as appropriate if sufficient statistical power was reported. The EPHPP tool has shown to be suitable for use in systematic reviews [45] and has fair inter-rater reliability and excellent agreement for the final rating [43].

## 3. Results

**Figure 1:**
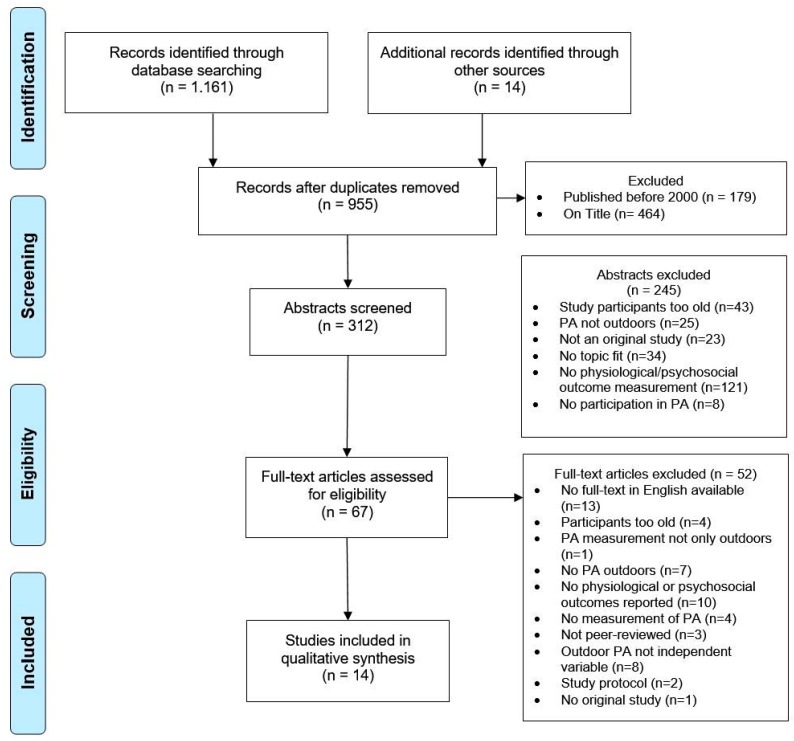
Flow diagram of the study selection process.

The study selection process is presented in Figure 1. A total of 1161 articles were identified in the four databases: 773 articles in Web of Science, 110 studies in PubMed, 139 studies in APA PsychNET, and 252 studies in ERIC. Through the snowball principle and contacts with our network, another 14 studies were added to the screening process. After the duplicates had been removed, a total of 955 studies remained for screening. At the end of the process, 14 articles representing 11 studies that met the inclusion criteria could be identified. One cohort study was published in three different articles [46,47,48]. Two of these articles differed only in the outcome whereas the study population and design were the same, the other article used a different design. Therefore, the two similar articles were treated as one in this review, the third one is listed separately.

Table 2 presents a summary of the studies included. Supplementary file 1 contains detailed quantitative results.

No studies published between 2000 and 2008 matched the inclusion criteria. Several study designs were represented: eight intervention studies (five crossover randomized controlled trials (RCTs), two nonrandomized controlled trials (CTs), one single group pre-post design), two prospective cohort studies, and three cross-sectional studies. Studies were conducted in the UK (*n* = 5), the US (*n* = 5), Australia (*n* = 2), and Japan (*n* = 1). The number of participants varied widely across studies, with a total of 9402 youth across studies. While the RCTs included between 14 and 86 study participants, the cohort studies included between 775 and 5238 participants. All of the studies looked at children aged 6–13 years, with two exceptions looking at four-year-olds [49] and 17-year-olds [47].

PA frequency, intensity, time, and type varied across studies. Looking at outdoor PA time, most of the intervention studies (*n* = 5) were short-term studies with one-time interventions taking 15–20 min [50,51,52,53,54]. The other intervention studies looked at effects over five days [55], four weeks [56], and four months [57]. In a prospective cohort study, participants were asked to report the amount of outdoor PA during an average week [47], cross-sectional studies asked for the amount of PA on an average day [58], during an average week [46,48], and during the last 24 h [49].

Looking at the frequency of outdoor PA, all short-term studies conducted a one-time intervention [50,51,52,53,54]. The longer intervention studies included daily activities during school recess [55,57], another study reported 274 outdoor PA bouts over four weeks for all participants together [56]. In a prospective cohort study and two cross-sectional studies, participants were asked to report their frequency of participation in outdoor PA, ranging from “very often” to “never” [59]. The other studies reported the total amount of outdoor PA, but not the frequency [46,47,48,58].

The type of PA varied widely across studies, including orienteering [54,55], ergometer cycling [50,53], walking [51], sports games and aerobic activities [46,47,48], and general PA outdoors without type specification [56,58,59].

Regarding outdoor PA intensity, two studies reported moderate PA levels [50,53], three studies reported moderate to vigorous physical activity (MVPA) [54,55,57], two other studies did not measure intensity, but assumed that the activities that could be chosen in the measurement met the MVPA intensity [46,47,48], and one study reported light PA and MVPA [49]. All other studies did not report PA intensity levels [51,52,56,58,59].

Looking at the reported outcomes, more psychosocial outcomes (*n* = 15) than physiological outcomes (*n* = 6) were examined. Psychosocially, self-esteem was the most assessed outcome, being measured in four studies [52,53,54,55,56]. Physiologically, blood pressure was the most assessed outcome, being measured two times [48,50]. All other outcomes were measured at most two times and with different measurement instruments. Therefore, pooling results and conducting a meta-analysis was deemed inappropriate. Regarding gender, one cohort study investigating the relationship between self-reported health and continuous participation in outdoor PA reported an increased odds ratio for the overall study population and boys, while the results for girls were not significant [59]. No other studies reported gender differences related to the outcome.

### 3.2. Quality of the Evidence

In Supplementary file 2, the results of the quality assessment are presented by study. Except for one moderate rating [51], all studies were rated as low quality. The poorest ratings were obtained in the categories of selection bias (*n* = 9, category mean rating = 2.86), blinding (*n* = 6; mean = 2.63), and data collection methods (*n* = 8; mean = 2.36). Reliability and validity of data collection methods [46,47,48,49,52,53,55] and blinding [50,52,54,55,56,57] were often not reported. The categories of confounders, blinding, and intervention integrity were not applicable in six studies due to their observational or cross-sectional design [46,47,48,49,58,59]. As most of the RCTs were crossover-trials with participants completing both conditions, no between-group differences could be responsible for the outcomes in both conditions, resulting in a strong rating (mean = 1.25) of the confounder section. Reporting of withdrawals and dropouts varied across studies (mean = 1.86).

None of the studies—except for one crossover RCT with complete data for all participants—considered the “intention to treat” principle in the statistical analysis, and only four studies [51,52,56,58] reported statistical power.

### 3.3. Evidence Synthesis

Table 3 provides an overview of the effectiveness and the psychosocial GE outcomes assessed, Table 4 provides an overview of the effectiveness and the physiological GE outcomes assessed.

#### 3.3.1. Effectiveness of GE

First, study outcomes and study characteristics will be summarized in terms of effectiveness.

*(1) Physical activity in the green condition was superior to the control condition for six outcomes*

Six studies reported a superior effect of PA in the green condition compared to the control condition for five psychosocial outcomes (attention, health-related quality of life, self-reported health, social support, and antisocial interactions) [47,51,56,57,59] and one physiological outcome (diastolic blood pressure (BP)) [50]. Each effect was only reported once. All studies were longitudinal studies (two crossover RCTs [50,51], one nonrandomized CT [57], one single-group pre-post study [56], and two cohort-studies [47,59]). In the single-group study [56], there was no control group, only comparison with baseline data, limiting the ability to draw causal conclusions. The crossover RCTs and single group study had a small number of participants, ranging from 14 to 27 [50,51,56], the nonrandomized CT had 437 participants [57] and the cohort-studies ranged from 775 to 5239 participants [47,59]. One study allowed only children diagnosed with attention deficit hyperactivity disorder (ADHD) as participants [51]. Participants of the intervention studies were all around the same age (9–12 years), while the cohort studies had baseline data of participants aged six and 12 years, respectively, with a follow-up period of five years [59] and six years [47]. The crossover RCTs applied short-term interventions of 15–20 min [50,51], the other intervention studies were four weeks [56] and four months [57]. One study was rated as moderate study quality [51], all other ones as low.

*(2) Physical activity is effective, but there was no difference between the green and the control condition for five outcomes*

Four studies reported an effect of PA on four psychosocial outcomes (self-esteem, vigor, tension, and fatigue) [50,52,53,54] and one physiological outcome (heart rate, [50]), but no differences between the green condition and the control condition could be observed. All studies were crossover RCTs, with sample size ranging between 14 and 86 children and with an average age of 10–13 years. PA in green and control conditions had a duration between 10 and 20 min. All studies were conducted by the same research group and were rated as low quality.

*(3) Physical activity does not show an effect in any condition/no differences between exposure and control group for 15 outcomes*

Ten studies reported no effect of PA or no difference between the green condition and control condition in terms of 11 psychosocial outcomes (self-esteem, vigor, tension, anger, depression, confusion, setting rating, PA self-efficacy and enjoyment, self-reported health) [50,51,52,53,55,56,58] and four physiological outcomes (systolic and diastolic BP, retinal diameter, sleep duration) [46,48,49,50]. Six were intervention studies (four RCTs [50,51,52,53] and one nonrandomized CT [55], and one single group pre-post design [56]), and three cross-sectional studies [46,48,49,58]. Sample size varied from 17 to 85 participants in the intervention studies and from 140 to 1765 in the cross-sectional studies. Participants of the intervention studies were between 9 and 12 years, and 4 to 13 years in the cross-sectional studies. Intervention duration varied between 15 min and five days in the intervention studies. Except for one study [56], all studies were rated as low quality.

*(4* *)* *Physical activity in the control condition is more effective than in the green condition for three outcomes*

Two studies, reporting one psychosocial outcome (health-related life quality [47]) and two physiological outcomes (diastolic and mean arterial BP, [46]), found a superior effect of PA in the control condition compared to the green condition. One study used a cohort design [47] and the other one a cross-sectional design [48]. Participants were aged seven years in the cross-sectional and 12 (baseline) and 17 (follow-up) years in the cohort study. Participants in both studies were part of the same study population and the studies were conducted by the same researchers. Study quality was rated low for both studies. 

#### 3.3.2. Overview of Psychosocial and Physiological Outcomes

In this section, the evidence is summarized based on psychosocial and physiological outcomes.

*(1) Psychosocial outcomes*

Fifteen different outcomes were reported in the psychosocial category. Except for self-esteem, all study outcomes were only assessed by one or two studies with a large variety of measurement instruments.

For attention (RCT) and antisocial interactions (nonrandomized CT), PA in the green condition showed stronger positive effects than PA in the control condition. Both studies were of low to moderate quality [51,57]. There was also a positive effect for PA outdoors and increased social support, but due to a single-group design, no conclusions can be drawn about superior effects compared to other settings [56].

When comparing children in the highest tertile of outdoor PA to the highest tertile of indoor PA, health-related qualify of life was higher for children being active outdoors, whereas comparing children in the lowest tertile of outdoor PA to the lowest tertile of indoor PA, children that were active indoors showed higher scores [47]. One cohort-study and one cross-sectional study looked at self-reported health, with the cohort study finding positive effects for frequent outdoor PA compared to infrequent outdoor PA [59], whereas the cross-sectional study found no significant associations [58].

Fatigue was reported as significantly higher post-exercise in two crossover RCTs, with no differences in the green and control condition [50,53]. Two studies reported results for vigor and tension. One study reported lower levels for each outcome post-exercise, the other study did not report any effect of exercise with no differences between green and control in both studies [50,53]. 

Self-esteem was assessed in four intervention studies with Rosenberg’s Self-Esteem scale [52,53,54,55]. The three RCTs with one, short single bout of exercise [52,53,54] reported increased self-esteem post-exercise, while the other RCT over five days did not find any effects on self-esteem with no differences between green and control condition in both studies [55].

For several outcomes, PA did not have an effect in any condition or was not different between green and control condition. This was true for several mood states [53], ratings of the environmental setting [51], PA enjoyment [52,56], and self-efficacy [56]. Except for PA enjoyment, each outcome was only reported in one study.

*(2) Physiological outcomes*

Six physiological outcomes were reported. For systolic BP, one crossover RCT found a positive effect for PA in the green condition compared to the control condition, while a cohort study found no difference when comparing youth being active outdoors to the ones being active indoors [48,50]. The same crossover RCT found a significant increase for heart rate post-exercise, but no differences between the conditions [50].

Looking at retinal diameter [46] and sleep duration [49], no effect was found in any condition. Each of these outcomes was only assessed in one study.

Contradictory results were found for diastolic BP. A crossover RCT did not find any effect on diastolic BP in any condition [50], while a cohort study did not find any differences in diastolic BP when comparing PA of children indoors and outdoors [48]. Interestingly, contradictory results were found within the same cohort study. While there was no difference in diastolic BP when comparing active children in- and outdoors in tertiles, the regression analysis only found a significant effect for indoor PA, but not for outdoor PA [48]. The same regression analysis also revealed a significant effect for PA indoors on mean arterial BP, but not for PA outdoors [48].

## 4. Discussion

Two purposes of this study were to provide an overview of the psychosocial and physiological outcomes of GE in children and adolescents and assess the effectiveness of GE. A total of 21 different outcomes were reported in the assessed studies. Each outcome was investigated by a maximum of two studies, except for self-esteem (four studies). When two studies assessed the same outcome, results were mostly contradictory, but comparisons were difficult due to study heterogeneity. Looking at the heterogeneity of results, quality of the evidence, and methodological considerations, the findings of this review are very similar to the review of Lahart and colleagues about the effects of GE in adults [28]. Recommendations for future research investigating outcomes of GE in children and adolescents will be outlined based on a more detailed discussion of the results.

### 4.1. Theoretical Background Considerations

Except for one study [51], none of the included studies provided a theoretical background to account for the assumed relationships between GE and outcomes. In other studies, Attention Restoration [60] and Stress Reduction Theory [61] have been applied [31,51,62,63]; however, based on these theories, benefits occur through contact with nature and are not dependent on PA levels. Thus, the underlying mechanisms regarding the interaction between the benefits of PA and nature exposure should be explored [25]. An ecological dynamic approach might be useful, assuming beneficial effects of GE due to nature’s action and immersive interaction possibilities, the holistic involvement of mind and body, and challenging situations [64]. Considering the lack of GE theories, qualitative research could provide valuable in-depth information to develop concepts, theories, and hypotheses which could then be tested with quantitative studies. A rigorous RCT with a two (PA or not) by two (natural environment or not) design and four intervention arms (PA in concrete environment, concrete exposure without PA, PA in natural environment, and nature exposure without PA) would allow more confident conclusions.

### 4.2. Assessed Outcomes Related to GE

For most outcomes, either no effect was found in GE and control group or effects were found for both groups. One reason for this could be the lack of theoretical background. For some outcomes, the assumption behind why the outcome should be different when exercising in the green compared to the non-green condition was not clear. Another explanation could be that it was often not clear if the measurement instruments are appropriate to measure the outcome of interest as validity and reliability were not reported. Thus, future studies should consider the theoretical background regarding GE and youth development to determine outcomes of interest and report validity and reliability of the measurement instruments.

At the same time, it is also important to investigate outcomes where exercising indoors might result in more positive effects than exercising outdoors, e.g., for feelings of safety and security. On three outcomes (health-related quality of life, diastolic and mean arterial BP), stronger effects were reported for the comparison group [47,48]. Looking at health-related quality of life, children in the lowest tertile of indoor PA reported better outcomes than children in the lowest outdoor PA tertile [47]. One reason could be that children who are less active might feel safer and more comfortable in an indoor environment with safety being related to PA [65]. Another explanation could be that children that prefer indoor activities do not like being exposed to weather variations. Regarding the better BP outcomes in the indoor PA group, the study’s authors explained the better effect of indoor activity with higher intensities during indoor PA compared to outdoor PA [48]. However, the inconsistent results of this study should be taken into consideration. Being aware of any superior effects of indoor PA and any deleterious effects of GE is especially important to adapt the setting accordingly for PA interventions.

### 4.3. Conceptual Considerations—What Is “Green”?

Pretty and colleagues defined GE as any exercise that is done in direct exposure to nature [26], referring to areas that include predominantly natural characteristics [64]. It is not clearly operationalized how many natural features of an area or the percentage of green in that area in order to be defined as “green”. Thus, “green” settings were inconsistent throughout the studies included, which has also been reported as a problem in GE studies with adults [28].

Natural environments offer various landscapes and features, therefore raising the question if different characteristics lead to different outcomes. Regarding self-esteem and mood in adults, stronger effects were found for waterside places, but no differences were reported between urban green space, countryside, wilderness, and woodlands [62]. Such questions are still open for children and adolescents and should be investigated as youth and adults differ in their environmental perceptions [66].

In two included studies, participants were exercising in a lab condition whilst viewing a natural or the control scenery on a screen [50,53]. Although this might already have positive health outcomes [27], the experience of nature is limited in several ways, such as the various action possibilities and immersive experiences [64]. Another perspective to look at GE comes from nature-based tourism, emphasizing PA in nature that focuses on enjoying natural attractions, stressing the conscious interaction with nature and not only nature experiences that occur in daily life. This is similar to Pretty’s level of involvement and participation in nature [27,67]. For adults, better effects of exercising during nature involvement and participation have been found compared to exercising in a control condition (built or indoor environment) for various outcomes, such as night sleep restoration [68], self-reported mental health [30], and directed attention and social interactions [69]. Moreover, outcomes of exercising during nature exposure in adults were also found for indirect ways of nature exposure. Positive effects of nature visuals and nature sounds included improved cognitive directed attention, mood and stress scores, compared to the control conditions [33,63]. While there are some positive results for adults, research on the different levels of nature exposure in youth is still limited. Especially when considering the amount of time children and adolescents spend on screen-based activities [70,71], applying a screen-based approach for GE might yield positive effects. Thus, it is not only important to investigate different natural features, but also to explore which effects different levels of exposure have on youth and how they differ from each other, such as watching nature video content during exercising on a treadmill, active transportation in nature, and going for a hike.

### 4.4. Characteristics of PA Outdoors

Looking at outdoor PA frequency and time in intervention studies, most studies reported a single bout of PA of up to 20 min, which is also commonly done when investigating GE in adults [28]. While GE already showed effects in adults after five minutes [62], it is unclear if this also applies to youth. Therefore, future studies should investigate GE over a longer time period to explore if GE effects depend on PA frequency and time. For example, one of the prospective cohort studies reported significant differences in health-related quality of life when comparing children in the highest tertile of outdoor PA to the highest tertile of indoor PA, while this was not true when comparing the lowest tertiles [47].

Although intensity levels have been reported in some studies, subgroup analyses have not been conducted to investigate if intensity levels impact the outcome. In adults, self-esteem showed the greatest improvements for moderate GE intensity, while mood had the best improvements when implementing light and vigorous GE [62]. These relationships are to be explored in future studies for the young age group.

Various types of activities have been reported in the included studies, with most of them being activities that can be implemented in daily life, such as walking, roller-skating, game activities, and general outdoor PA without type specification [46,47,48,51,56,58,59]. Nature offers various action possibilities with a challenging character, such as rock climbing and mountain-biking, that are also called outdoor adventures [64]. Compared to daily PA activities, these activities include additional components like a small group setting, an unfamiliar physical environment, and challenges allowing mastery experiences [72]. While this is worth investigating, it should be carefully considered if the mechanisms leading to outcomes such as changes in a person’s self-concept, skills, and attitudes [72] are due to GE, the adventurous character or a mixture of both. For children, outdoor play is also a possible type of GE, however, PA levels vary widely during outdoor play [73] so it cannot be considered automatically as GE without measurement, nor is it clear if all playgrounds could be considered green.

Looking at the measurement of GE, most of the intervention studies included in this review used device-based measurements with accelerometer or heart rate monitoring while the researcher reported the setting the participants were exposed to. Another method is the use of validated observation instruments such as SOPLAY and SOPARC [57], requiring the researcher’s presence for measurement. To assess PA levels in a spatial context objectively, one way would be combining accelerometer, GPS, and GIS data [74,75]. Several studies included in this review have also used self-report measures such as questionnaires and diaries. However, none of these studies reported validity and reliability of these instruments to assess outdoor PA in children and adolescents. Therefore, development of a valid and reliable self-report GE instrument would be helpful, e.g., when assessing GE in a large number of children or when resources are limited.

### 4.5. Study Population and Sample Size

Except for two studies looking at preschool children [49] and older adolescents [47], all studies focused on children 6–13 years old. Due to the nature of youth development, evidence that is valid for one age group might not be applicable to another. To allow conclusions about outcomes of GE across childhood and adolescence, future studies should include different age groups of youth in their study population. Except for two studies with ADHD-children [51] and samples with some overweight participants [50,56], none of the samples had a clinical background. When ethnicity was reported, most participants were Caucasian [46,47,48,49] or Asian [59]. Thus, future studies should investigate GE in young participants across different ethnicities, cultures, backgrounds, and settings.

To determine the appropriate sample size, one cross-sectional and three intervention studies [51,52,53,58] provided a power analysis. Especially, looking at the small sample sizes in some intervention studies [50,51,53,54,56], which is also a problem in adults [28], future studies should include larger sample sizes to detect small effects and to avoid type II errors [76].

### 4.6. Quality Assessment

All but one [51] study received a weak rating based on the EPHPP tool. These results are comparable to the review of Lahart and colleagues, who also rated the GE study quality in adults as weak [28]. However, the quality assessment results for this review should be viewed with caution, considering the categories and the focus of the quality assessment tool. The aim of the included studies was to explore the relationship between health and youth’s GE, thereby focusing less on representative samples. Thus, selection bias might not be as important as other categories of the EPHPP tool. Blinding should also be considered carefully as it is not possible to blind participants to the environmental condition they are exposed to. One study blinded participants to the research question [51], but this might not be possible in other studies due to ethical considerations. Another option is to assess blinding in the context of the outcome measurement: A meta-epidemiological study revealed that lack of blinding only increases the risk of bias for subjective, but not objective outcome measurements [77]. 

For future systematic reviews in this area, a quality assessment tool with a less clinical focus would be helpful. This tool may include the categories of the EPHPP tool, but different categories should receive a different weight, such as focusing less on selection bias and blinding.

### 4.7. Study Limitations

This systematic review does not come without limitations. Regarding the included studies, several weaknesses have already been outlined, comprising limited comparability due to heterogeneity of study results and study designs as well as the low quality of the evidence. Another aspect to consider is that in some studies, outdoor PA instead of GE had been investigated, so that it was not clear how much green features were around the participants during PA.

As is common in systematic reviews, the first screening of studies to be included was based on title alone, so that some studies might have been overlooked. The search was limited to studies published after 2000. Including studies before that year might have helped finding more consistent outcomes, even though GE had not been defined yet. The terms included for the study search were phrased to identify studies of nonclinical populations. To explicitly include GE studies in a therapeutic and medical context, some additional search terms would have to be added.

## 5. Conclusions and Future Directions

GE does not have negative effects for children and adolescents compared to exercising in a built or an indoor environment. There are some indications that PA in nature-based environments has beneficial effects; however, due to the heterogeneity of study results that limits comparisons for specific outcomes and small sample sizes, it is premature to draw conclusions. Considering these findings in the context of the previous systematic review about GE in adults [28], the following recommendations can be applied to children, adolescents, and adults.

Future research should investigate the underlying effects and mechanisms of GE in order to establish GE theories which can be used to determine possible GE outcomes. Especially, when establishing GE theories for children and adolescents, a qualitative approach using for example Grounded Theory [78] could be helpful. Another way would be to review current literature on possible mechanisms of both PA and nature contributing to health and combining them in a theoretical framework. While it is important to have a theory for the GE field that includes both PA and nature, it is also essential to test this theory with quantitative methods so that it can be adapted if necessary and applied to future interventions. Both short- and long-term outcomes of interest should be investigated across different cultures and age groups in childhood and adolescence and specific outcomes explored across frequencies, intensities, time, and type of GE. To investigate short-term effects, an ambulatory assessment approach could be promising that allows capturing data on nature, PA, and outcomes of interest in real-time and natural settings of study participants, thus assessing outcomes of GE in daily life [79,80]. For long-term outcomes, using a cohort-study design where GE is measured from childhood over adolescence to adulthood would be helpful to assess outcomes of long-term participation in GE. To test causalities, RCTs with a longer time period could yield valuable results. In such designs, it would be important to expose participants to nature over several weeks or months on a continuous basis (e.g., twice a week) and a meaningful amount of time (e.g., one hour of GE) to investigate long-term effects.

## Figures and Tables

**Table 1:** Study selection criteria.

PICOS	Eligibility Criteria
*Population*	Study participants younger than 18 years
*Intervention*	Any PA/exercise conducted in nature (independent variable) Measurement of PA/exercise
*Comparison*	No firm comparison group determined
*Outcomes*	Any psychosocial or physiological outcome measured and reported Psychosocial outcomes: individual’s social and psychological aspects, including, but not limited to cognitions, emotions, and mental health [39,40] Physiological outcomes: bodily changes due to stimuli response [41]
*Study Design*	No limitations regarding the study design

**Table 2:** Characteristics of the included studies.

Author/Year	Study Design	Participants and Country	Type of Green Exercise and Procedures	Outcome Variable(s)	Measurement Instrument(s)	Results	Quality Assessment
Barton et al. (2015) [55]	Crossover RCT	52 boys and girls Mean age: 9 years UK	Intervention and control condition in both urban and rural school, available during lunch time break (55 min) at both schools Intervention: five days of nature-based orienteering (NBO) Control: five days provision of playground sports equipment (PSE) on not-green playground during recess	Self-esteem (SE)	Accelerometer Rosenberg SE scale	○ = SE	Weak SB: 3, SD: 2, C: 2, B: 3, DCM: 3, WD: 3
Duncan et al. (2014) [50]	Crossover RCT	14 children (50% female) Mean age: 10 years UK	Two 15 min bouts of cycling at in two lab conditions Intervention: green condition (viewing a film of cycling in a forest) Control condition: viewing a black screen	Blood pressure (BP) Heart rateMood state response	Automated oscillometric device Heart rate monitor Fatigue, tension and vigor subscales of Brunel Mood State Inventory	↓↓ systolic BP 15 min post-exercise ○ = systolic BP or diastolic BP immediately post-exercise ↑ = HR immediately and 15 min. post-exercise ↑ fatigue score ↓ = vigor score ○ = tension	Weak SB: 3, SD: 1, C: 1, B: 3, DCM: 1, WD: 1
Faber Taylor & Kuo (2009) [51]	Crossover RCT	17 children with (12% female) attention deficit hyperactivity disorder (ADHD) Mean age: 9 years USA	20 min guided walk in three different settings Intervention: park (green area) Control 1: residential area Control 2: downtown area	Attention Child’s rating of settings	Digit Span Backwards (DSB) three-point scale for children to rate walk as fun, relaxing, interesting, scary, boring, weird, and/or uncomfortable	↑↑ Digit Span Backwards score post-intervention ↑↑ fun rating = ratings for relaxing, interesting, scary, boring, weird, and/or uncomfortable	Moderate SB: 3, SD: 1, C: 1, B: 1, DCM: 1, WD: 2
Flynn et al. (2017) [56]	One group pre-post	27 children in 16 families (51.9% female) Mean age: 11 years USA	Four-week outdoor PA family intervention	PA self-efficacy PA enjoyment PA social support	Weekly PA activity logs (filled in by parents) Self-administered survey for children on PA enjoyment, SE, and social support	○ PA enjoyment and self-efficacy ↑ PA social support	Weak SB: 3, SD: 2, C: 1, B: 3, DCM: 2, WD: 3
Gopinath, Baur et al.; Gopinath, Hardy et al. (2011) [46,48]	Cross-sectional	1765 children (48.3% female) [48], 1492 children (49.3% female) [46] Mean age: 7 years Australia	Comparison of children Exposure: low, middle and high tertile of outdoor PA Control: low, middle and high tertile of indoor PA Linear associations between BP and indoor/outdoor PA	Retinal Arteriolar and venular Diameter Systolic BP Diastolic BP Mean arterial BP	Questionnaire on PA (proxy-report through parents) Retinal photography Automated sphygmomanometer	= retinal arteriolar and venular diameter = systolic and diastolic BP Linear association between indoor PA and ↓ diastolic BP and ↓ mean arterial BP, ○ systolic BP ○ outdoor PA and BP outcome	Weak SB: 2, SD: 3, C: N/A, B: N/A, DCM: 3, WD: 1
Gopinath et al. (2012) [47]	Prospective cohort study	Cross-sectional: 1094 adolescents (56.1 % female) longitudinal: 775 children and adolescents Mean age: 12 years at basline, 17 years at follow-up Australia	five-year cohort study. Comparison of children cross-sectionally (at follow-up) and longitudinally. QoL was only measured at follow-up. Comparison of children Exposure: low, moderate and high tertile of outdoor PA Control: low and moderate-high tertile of indoor PA	Health related quality of life (QoL)	Questionnaire on PA Pediatric Quality of Life Inventory 4.0	↑ QoL in control group in low tertiles ↑↑ QoL comparing high and moderate-high tertiles	Weak SB: 2, SD: 2, C: N/A, B: N/A, DCM: 3, WD: 3
Hammond et al. (2011) [58]	Cross-sectional	140 parents (84% female) of children between 6 and 13 years USA	One-time questionnaire accessed through parents (proxy-reporting) to report about children’s health problems and PA in two settings Exposure: PA outdoors Control: PA indoors	Health problems	Health inventory Questions on outdoor and indoor organized activities and sports	○ = health problems (body pain/discomfort, trouble sleeping, repeated upset stomach, feeling tired/having low energy)	Weak SB: 3, SD: 3, C: N/A, B: N/A, DCM: 3, WD: 1
Liu et al. (2015) [59]	Prospectivecohort study	5238 children (52.2% female) Mean age: 6 years at baseline, 12 years at follow-up Japan	six-year cohort study (baseline at age 6). Questionnaire on frequency of outdoor PA and self-reported health at baseline and follow-up. Exposure: frequent outdoor PA Control: infrequent outdoor PA	Self-reported health	Self-report questionnaire about outdoor PA Dartmouth Primary Care Cooperative project Charts (Self-report instrument about health)	↑↑ self-reported health at baseline and follow-up	Weak SB: 2, SD: 2, C: N/A, B: N/A, DCM: 3, WD: 3
Parsons et al. (2018) [49]	Cross-sectional study	447 children (51.5% female) Mean age: 4 yearsUSA	Data collection on sleep and PA indoors and outdoors in child-care centers Exposure: outdoors Control: indoors	Sleep duration	Sleep diary filled in by parents and child care center staff Accelerometer Observation of outdoor and indoor PA through child care center staff	○ = sleep duration	Weak SB: 2, SD: 3, C: N/A, B: N/A, DCM: 3, WD: 1
Raney et al. (2019) [57]	Nonrandomized controlled trial	437 children (51.1% female) 5^th^ grade students USA	Outdoor PA in school playgrounds during 20 min recess Intervention: playground greening at one school Control: no greening	Antisocial interactions	Accelerometers System for Observing Play and Leisure Activity in Youth (SOPLAY) System for Observing Children’s Activity and Relationships During Play (SOCARP)	↓↓ of physical and verbal conflicts after four months ↓ of minutes spent alone and ↑ increase of minutes spent in small groups in intervention group	Weak SB: 3, SD: 1, C: 3, B: 3, DCM: 1, WD: 3
Reed et al. (2013) [52]	Crossover RCT	86 boys and girls Age: 11–12 years UK	Running over 1.5 miles in two settings; participants engaged in both conditions Intervention: green setting Control: urban nongreen setting	SE Exercise enjoyment Perceived exertion	PA questionnaire for adolescents Fitnessgram Pacer Test Rosenberg SE Scale Ratings of Perceived Exertion Scale	↑ = SE = ratings of perceived exertion and enjoyment	Weak SB: 3, SD: 3, C: N/A, B: N/A, DCM: 3, WD: 1
Wood et al. (2013) [53]	Crossover RCT	25 children (56% female) Age: 11–12 years UK	Laboratory condition: All participants engaged in two constant load tests on a cycle ergometer (10 min) whilst viewing two types of picture series Intervention: natural environment pictures Control: built environment pictures	SE Mood	Rosenberg SE scale Adolescent Profile of Mood States Questionnaire (PMSQ)	↑ = SE and fatigue ↓ = tension ○ = depression, anger, vigor and confusion	Weak SB: 3, SD: 1, C: 1, B: 2, DCM: 3, WD: 1
Wood et al. (2014) [54]	Crossover RCT	60 children (50% female) Mean age: 13 years UK	Participants engaged in two orienteering courses (20 min, respectively) Intervention: natural environment Control: built environment	SE	Accelerometer Rosenberg SE scale	↑ = SE	Weak SB: 3, SD: 1, C: 1, B: 3, DCM: 2, WD: 3

Table legend: ↑ increase; ↑↑ stronger increase/effect in intervention/exposure group compared to control group; ○ no effect /association; = no differences between intervention/exposure and control group; ↓ decrease; ↓↓ stronger decrease/effect in intervention/exposure group compared to control group; If = is combined with another symbol (e.g., ↑=), this means that both intervention/exposure and control group had the same effect; SE = self-esteem; BP = blood pressure; SB = selection bias, SD = study design, C = Confounders, B = Blinding, DCM = Data collection methods, WD = Withdrawals/Dropouts, N/A = Not applicable.

**Table 3:** Effectiveness and psychosocial outcomes of green exercise (GE).

Psychosocial Outcome	Stronger/only Effect Intervention/Exposure Group	Effect both in Intervention and Control Group	No Effect neither in Intervention or Control Group/ No Differences between Exposure and Control Group	Stronger/Only Effect in Control Group
Self-esteem		↑ [52,53,54]	[55]	
Fatigue		↑ [50,53]		
Vigor		↓ [50]	[53]	
Tension		↓ [53]	[50]	
Anger			[53]	
Depression			[53]	
Confusion			[53]	
Attention	↑ [51]			
Setting rating			[51]	
PAself-efficacy			[56]	
PA enjoyment			[52,56]	
Social support	↑ [56]			
Health-related quality of life	↑ [47]			↑ [47]
Self-reported health	↑ [59]		[58]	
Antisocialinteractions	↓ [57]			

Table legend: ↑ positive association; ↓ negative association.

**Table 4:** Effectiveness and physiological outcomes of GE.

Physiological Outcome	Stronger/only Effect Intervention/Exposure Group	Effect both in Intervention and Control Group	No effect neither in Intervention or Control Group/ No Differences between Exposure and Control Group	Stronger/Only Effect in Control Group
Systolic BP	↓ [50]		[48]	
Diastolic BP			[48,50]	↓ [48]
Mean arterial BP				↓ [48]
Heart rate		↑ [50]		
Retinal diameter			[46]	
Sleep duration			[49]	

Table legend: ↑ positive association; ↓ negative association.

## Supplementary Materials

The following are available online at https://www.mdpi.com/1660-4601/16/21/4266/s1, Table S1: Test statistics and descriptives, Table S2: Quality assessment with the EPHPP tool.
