# Supplementary material for: Psychosocial and Physiological Health Outcomes of Green Exercise in Children and Adolescents—A Systematic Review
Source: Int J Environ Res Public Health. 2019 Nov 2;16(21):4266. doi: 10.3390/ijerph16214266 (PMC6862643; doi:10.3390/ijerph16214266)
Supplement: Supplementary file 1 [file ijerph-16-04266-s001.zip › Supplementary_File_1.pdf]

Additional file 1: Test statistics and descriptives

Author/ year	Results		
Barton et al. (2015)	No effects on the SE change score due to intervention type, school location or interaction of both (all p > 0.05). Pre-intervention SE score affected SE score change (F[1,77] = 25.09; p < .01).		
		SE change scores	
		**Nature-based orienteering**	**Playground sports equipment**
	Urban school	2.16 ± 5.81 [0.68–5.16]	2.33 ± 6.69 [-0.65–3.66]
	Rural school	0.59 ± 3.33 [-0.97-3.19]	0.78 ± 5.18 [-1.68-2.46]
Duncan et al. (2014)	No interaction effects (condition X time) or main effects (all p > 0.05) for diastolic BP. For systolic BP, no significant interaction or main effects immediately post-exercise (p > .05). Significant condition X time interaction for systolic BP 15 minutes post-exercise (F [2,26] = 3.49, p = 0.04, Pɳ2 = 0.212) with systolic BP significantly lower after green exercise. No significant condition X time interaction for heart rate, no main effect for condition (both p > .05). Significant main effect with HR being higher immediately and 15-min. post-exercise (F [2,26] = 47.19, p < .01, Pɳ2 = 0.784). Significant mood scale X time (F [2,12] = 48.6, p < .01, Wilks' Lambda = 0.11, Pɳ2 = 0.89). Post-exercise, significantly higher fatigue scores and significantly lower vigor scores (Bonferroni corrected p = 0.001). Scores for tension were not significantly different (p > 0.05).		
		**Green exercise**	**Control condition**
		Diastolic BP (mm Hg)	
	Pre	m=69.5 [64.2-74.8]	m=64.7 [60.4-68.9]
	Immediately post	m=68.4 [61-75.8]	m=70.6 [62.9-78.2]
	15 minutes post	m=66.6 [62.6-70.5]	m=64.4 [58.8-68.3]
		Systolic BP (mm Hg)	
	Pre	m=103.9 [99.5-108.2]	m=102.2 [98.4-105.9]
	Immediately post	m=111.2 [108.1-117.3]	m=112.7 [103-121.4]
	15 minutes post	m=97.2 [93.9-100.4]	m=102.7 [99.1-108.6]
		Heart rate (bpm)	
	Pre	m=81 [76-84]	m=83 [76-89]
	Immediately post	m=102 [95-108]	m=106 [101-109]
	15 minutes post	m=93 [89-97]	m=94 [86-101]
		Mood state	
	Fatigue pre	m=39.3 [39-42]	m=39.8 [37.8-42.2]
	Fatigue post	m=45.3 [43.5-49.2]	m=47 [44.5-50]
	Vigor pre	m=52.1 [49-55]	m=51.5 [48.6-54.6]
	Vigor post	m=45 [42.1-49]	m=44.4 [40.6-48.6]
	Tension pre	m=45.8 [43.1-48]	m=45 [42.9-47]
	Tension post	m=44.4 [42.9-46.3]	m=44.5 [43-45.6]
Faber Taylor & Kuo (2009)	DSB performance dependent on setting (F [2,16] = 4.72, p < .05). No significant DSB performance differences between neighborhood and downtown settings (p > .05). DSB scores better after park walk (Fisher's PLSD d = .71, p < .01), and downtown walk (Fisher's PLSD d = .59, p < .05).<br>Park setting significantly higher on fun (t [13] = 2.39, p < .05). No other significant rating differences (all p > .05).		
		DSB performance mean scores after walk in each setting	
	Neighborhood	m=3.71 ± 1.21	
	Downtown	m=3.82 ± 1.07	
	Park	m=4.41 ± 1.18	
Flynn et al. (2017)	A request was sent out to the other for the exact test statistics, but no reply was obtained. No significant differences for PA self-efficacy and enjoyment. At follow-up, children reported increased frequency of someone having performed a physical activity or played a sport with them (median score, 2; range, 2–3) and that someone had provided transportation to a place where they could do physical activities or sports (median score, 2; range, 1–3).		

Gopinath, Baur et al.; Gopinath, Hardy et al. (2011)	No significant differences between the tertile groups of indoor and outdoor PA for retinal arteriolar and venular diameter and systolic and diastolic BP.<br>Linear associations between BP and outdoor and indoor PA: No significant effect of outdoor PA on any BP measures (systolic / diastolic / mean arterial BP). Significant effect of indoor PA on diastolic BP and mean arterial BP.

	Outdoor PA	Indoor PA
	Retinal arteriolar caliber (μm)	
Low tertile	m=162.5 [160.9-164.1]	m=163.3 [161.6-164.9]
Moderate tertile	m=163.0 [161.0-165.1]	m=164.7 [162.0-167.4]
High tertile	m=164.7 [163-166.5]	m=162.4 [161.0-163.8]
	Retinal venular caliber (μm)	
Low tertile	m=229.7 [227.2-230.8]	m=229.0 [227.2-230.8]
Moderate tertile	m=228.8 [226.6-231.1]	m=228.8 [226.6-231.1]
High tertile	m=229.3 [226.3-230.4]	m=228.2 [226.2-230.2]
	Systolic BP (mm Hg)	
Low tertile	m=100.8 [99.1-102.4]	m=101.2 [99.6-102.8]
Moderate tertile	m=100.8 [99.4-102.3]	m=100.8 [99.5-102.1]
High tertile	m=100.9 [99.4-102.5]	m=99.9 [98.3-101.5]
	Diastolic BP (mm Hg)	
Low tertile	m=63.0 [60.8-65.2]	m=62.6 [60.7-64.5]
Moderate tertile	m=62.9 [61.1-64.6]	m=62.4 [60.2-64.5]
High tertile	m=61.5 [59.5-65.5]	m=62.0 [60.2-63.9]
	Multiple Regression on BP outcomes (mm Hg)	
Systolic BP	β=.38 (SE: .34), p > .05	β= -1.76 (SE: 1.26), p > .05
Diastolic BP	β= -.80 (SE: .42), p > .05	β= -2.35 (SE: .73), p < .01
Mean arterial BP	β= -.41 (SE: .33), p > .05	β= -2.15 (SE: .75), p < .01

Gopinath et al. (2012)	For indoor PA, the moderate- and high-PA tertiles were only presented summarized.<br>Higher QoL total score, physical, psychosocial, emotional, social and school scores in low tertiles of indoor PA compared to low tertiles of outdoor PA<br>Higher QoL total score, physical, psychosocial, emotional, social and school scores in high tertiles of outdoor PA compared to moderate-high tertiles of indoor PA<br><br>*Note: The CIs were not presented in the original study but calculated by the authors of this review to allow for comparisons between outdoor and indoor PA in terms of health-related life quality. Mean differences were considered as significant when CIs were not overlapping.*

	Outdoor PA	Indoor PA
	Quality of life total score	
Low tertile cross-sectional (CS)	m=79.65 [79.54-79.76]	m=80.23 [80.77-80.89]
Moderate tertile CS	m=79.84 [79.71-79.97]	m=79.40 [79.28-79.52]
High tertile CS	m=81.84 [81.73-81.95]	
Low tertile longitudinal (LT)	m=78.68 [78.25-79.11]	m=80.75 [80.10-81.40]
Moderate tertile LT	m=79.30 [78.83-79.77]	m=79.66 [79.17-80.15]
High tertile LT	m=83.12 [82.75-83.49]	
	Physical score	
Low tertile CS	m=89.23 [89.11-89.35]	m=90.60 [90.62-90.76]
Moderate tertile CS	m=89.61 [89.47-89.75]	m=90.33 [90.19-90.47]
High tertile CS	m=92.72 [92.59-92.85]	
Low tertile LT	m=89.03 [88.60-89.46]	m=94.35 [93.62-95.08]
Moderate tertile LT	m=89.11 [88.64-89.58]	m=93.35 [92.80-93.90]
High tertile LT	m=95.11 [94.74-95.48]	
	Psychosocial score	
Low tertile CS	m=75.00 [74.87-75.13]	m=76.07 [76.00-76.14]
Moderate tertile CS	m=75.04 [74.89-75.19]	m=74.05 [73.90-74.20]

	High tertile CS	m=76.56 [76.42-76.70]		
	Low tertile LT	m=89.03 [88.60-89.46]	m=73.97 [73.16-74.78]	
	Moderate tertile LT	m=89.11 [88.64-89.58]	m=72.91 [72.67-73.15]	
	High tertile LT	m=95.11 [94.74-95.48]		
		Emotional score		
	Low tertile CS	m=72.65 [72.48-72.82]	m=73.75 [73.65-73.85]	
	Moderate tertile CS	m=72.40 [72.21-72.59]	m=71.06 [70.87-71.25]	
	High tertile CS	m=73.97 [73.80-74.14]		
	Low tertile LT	m=70.16 [69.46-70.86]	m=69.46 [68.36-70.56]	
	Moderate tertile LT	m=69.61 [68.85-70.37]	m=68.00 [67.19-68.81]	
	High tertile LT	m=73.47 [72.87-74.07]		
		Social score		
	Low tertile CS	m=89.05 [88.92-89.18]	m=90.84 [90.77-90.91]	
	Moderate tertile CS	m=88.67 [88.52-88.82]	m=88.63 [88.48-88.78]	
	High tertile CS	m=92.62 [92.48-92.76]		
	Low tertile LT	m=88.21 [87.74-88.68]	m=89.98 [89.17-90.79]	
	Moderate tertile LT	m=90.08 [89.57-90.59]	m=89.43 [88.82-90.04]	
	High tertile LT	m=93.54 [93.14-93.94]		
		School score		
	Low tertile CS	m=64.11 [63.92-64.30]	m=64.41 [64.30-64.52]	
	Moderate tertile CS	m=64.99 [64.78-65.20]	m=63.56 [63.35-63.77]	
	High tertile CS	m=64.03 [63.83-64.23]		
	Low tertile LT	m=63.30 [62.54-64.06]	m=63.32 [62.14-64.50]	
	Moderate tertile LT	m=65.16 [64.33-65.99]		
	High tertile LT	m=65.64 [65.01-66.27]	m=63.00 [62.13-63.87]	
Hammond et al. (2011)	No significant correlations neither between outdoor organized activities / sports and health problems or indoor organized activities / sports (all p > .05)			
		**Outdoor organized activities / sports**	**Indoor organized activities / sports**	
	Body pain / discomfort	r=.05, p>.05	r= -.05, p>.05	
	Trouble sleeping	r= -.13, p>.05	r= -.01, p>.05	
	Repeated upset stomach	r= -.04, p>.05	r= -.04, p>.05	
	Feeling tired / having low energy	r= -.04, p>.05	r= -.04, p>.05	
Liu et al. (2015)	Children who took part in outdoor PA had higher proportion of good self-reported health than those without. For females, only significant health differences between frequent and infrequent outdoor PA at age 12. Participation in outdoor PA at both ages 6 and 12 years is associated with a higher likelihood of good self-reported health (OR= 1.27[1.08, 1.50]) compared with those who did not like or participate in this at only one or at neither age. OR 1.47[1.14-1.89] for persistent outdoor participation in boys, no significant different likelihood for persistent outdoor PA in girls (OR= 1.14 [.92-1.42]).			
		Good self-reported health		
		**Frequent outdoor PA**	**Infrequent outdoor PA**	
	Males 6 years	85.5%	79.7%	*p<.01*
	Males 12 years	86.5%	81.5%	*p<.01*
	Females 6 years	81.0%	80.5%	*p>.05*
	Females 12 years	82.5%	78.7%	*p<.05*
	Both ages	82.3%	79.1%	*p<.05*
Parsons et al. (2018)	No associations between napping, nighttime sleep duration and total sleep with PA time indoors or outdoors.			
		**Outdoor PA**	**Indoor PA**	
	Nighttime sleep (hours)	m=9.69 ± .97<br>β=.02 (SE=.04), p>.05	m=9.69 ± .97<br>β=.07 (SE=.05), p<.05	
	Nighttime and nap sleep (hours)	m=11.20 ± 1.03<br>β= -.01 (SE=.06), p>.05	m=11.20 ± 1.03<br>β= .10 (SE=.06), p>.05	
	Napped at center	OR 1.10 [.67-1.81]	OR .88 [.61-.125]	
	Bedtime after 9 pm	OR 1.06 [.9-1.26]	OR .81 [.7-.94]	

Raney et al. (2019)	Condition X time interaction for antisocial behavior (F[2,998]=10.28, p<0.01) with physical and verbal conflict rates decreasing below pre-greening rates after 4 months at the experimental location. Significant decrease in minutes spent alone (mean difference= −2.2 [1.7−2.7], p<0.01) and significant increase in the number of minutes spent in small groups (mean difference=1.7 [0.9-2.6], p<0.01) at experimental location.			
		Number of antisocial interactions during 20 min. recess		
		**Greening location**	**Control location**	
	Pre-greening	m=3.5 [2.5-4.5]	m=3.6 [3.2-4.0]	
	Post-greening	m=4.6 [3.0-6.2]	m=2.9 [1.5-4.3]	
	4 month follow-up	m=1.8 [1.0-2.6]	m=3.2 [2.6-3.8]	
Reed et al. (2013)	There was a significant main effect for exercise on self-esteem (F[1,74]=12.2, p<.01), but no main effect for exercise condition (F[1,74]=0.02, p>.05) and no interaction (F[1,74] = 0.13, p>.05). No significant differences between green and control exercise in terms of enjoyment (t[75] = 0.43, p>.05), ratings of perceived exertion (t[75] = 0.11, p>.05) or change in self-esteem (t[75] = 0.13, p>.05).			
		**Green Exercise**	**Control condition**	
	Δ Self-esteem	.9 ± 2.6	.5 ± 4.2	*p>.05*
	Ratings of perceived exertion	m=13.5 ± 3.6	m=13.6 ± 3.6	*p>.05*
	Enjoyment	m=89.2 ± 31.4	m=83.6 ± 34.7	*p>.05*
Wood et al. (2013)	Significant main effect on SE due to PA (F[1]=6.10, p<.05) but not due to the environmental viewing condition (p>.05). For mood, no significant effect of viewing different environmental conditions (p>.05). Main effect for mood changes and PA participation (F[6]=5.29, p<.01). PA resulted in significant increase in fatigue (F[1]=8.11, Bonferroni corrected p<.0083) and decrease in tension (F[1]=11.56, Bonferroni corrected p<.0083). No other significant pre-post changes on other mood sub-scale (all p > .0083). No significant main effect for total mood disturbance due to participation in PA or environmental viewing condition (all p>.05).			
		**Green Exercise**	**Control condition**	
	Tension pre	m=49.7 ± 7.9	m=50.3 ± 9.3	
	Tension post	m=50.3 ± 9.3	m=44.7 ± 6.1	
	Depression pre	m=45.8 ± 4.0	m=45.0 ± 3.2	
	Depression post	m=45.9 ± 4.3	m=45.3 ± 3.7	
	Anger pre	m=46.6 ± 6.1	m=46.7 ± 5.6	
	Anger post	m=47.0 ± 9.4	m=46.9 ± 6.7	
	Vigour pre	m=53.4 ± 9.1	m=50.3 ± 8.7	
	Vigour post	m=49.8 ± 9.4	m=48.6 ± 9.3	
	Fatigue pre	m=47.6 ± 10.1	m=46.6 ± 9.5	
	Fatigue post	m=52.4 ± 9.5	m=52.6 ± 10.2	
	Confusion pre	m=46.9 ± 5.6	m=46.4 ± 5.5	
	Confusion post	m=45.2 ± 4.5	m=45.2 ± 5.1	
Wood et al. (2014)	No significant interaction for time X environmental condition for SE. No significant main effect due to the orienteering environment (all p>0.05). Significant time main effect for SE (F[1,49]= 5.24; p<.05, n_p=0.1).			
		Self-esteem scores		
		**Green Exercise**	**Control Condition**	
	Pre	m=31.5 [30.2-32.3]	m=31.4 [30.1-32.1]	
	Post	m=31.9 [30.6-32.7]	m=32.4 [31.1-33.3]	
