# Supplementary material for: Psychosocial and Physiological Health Outcomes of Green Exercise in Children and Adolescents—A Systematic Review
Source: Int J Environ Res Public Health. 2019 Nov 2;16(21):4266. doi: 10.3390/ijerph16214266 (PMC6862643; doi:10.3390/ijerph16214266)
Supplement: Supplementary file 1 [file ijerph-16-04266-s001.zip › Supplementary_file_2.pdf]

Supplementary file 2: Quality assessment with the EPHPP tool

Category and Global rating

Study	*Selection Bias*	*Study Design*	*Confounders*	*Blinding*	*Data Collection Methods*	*Withdrawal / Dropouts*	***Overall Rating***
Barton et al. (2015)	3	2	1	3	3	3	**3**
Duncan et al. (2014)	3	1	1	3	1	1	**3**
Faber Taylor & Kuo (2009)	3	1	1	1	1	2	**2**
Flynn et al. (2017)	3	2	1	3	2	3	**3**
Gopinath, Baur et al. / Gopinath, Hardy et al. 2011	2	3	N/A	N/A	3	1	**3**
Gopinath et al. (2012)	2	2	N/A	N/A	3	3	**3**
Hammond et al. (2011)	3	3	N/A	N/A	3	1	**3**
Liu et al. (2015)	2	2	N/A	N/A	3	3	**3**
Parsons et al. (2018)	2	3	N/A	N/A	3	1	**3**
Raney et al. (2019)	3	1	3	3	1	3	**3**
Reed et al. (2013)	3	1	1	3	3	1	**3**
Wood et al. (2013)	3	1	1	2	3	1	**3**
Wood & Smyth (2014)	3	1	1	3	2	3	**3**

Intervention Integrity and Analyses

	Intervention Integrity			*Analyses*			
	% participants receiving allocated exposure	*Consistency measured*	*Likely that subjects received an unintended intervention*	*Unit of allocation*	*Unit of analyses*	*Statistical methods appropriate*	*Analyses by allocation status rather than actual intervention received*
Barton et al. (2015)	80-100	Can't tell	Can't tell	Individual	Individual	No	No
Duncan et al. (2014)	80-100	Yes	Can't tell	Individual	Individual	No	Yes
Faber Taylor & Kuo (2009)	80-100	Yes	Can't tell	Individual	Individual	Yes	No
Flynn et al. (2017)	80-100	Yes	Can't tell	Individual	Individual	Yes	No
Gopinath, Baur et al. 2011	N/A			Individual	Individual	No	No
Gopinath, Hardy et al. (2011)	N/A			Individual	Individual	No	No
Gopinath et al. (2012)	N/A			Individual	Individual	No	No
Hammond et al. (2011)	N/A			Individual	Individual	Yes	N/A
Liu et al. (2015)	N/A			Individual	Individual	No	No
Parsons et al. (2018)	N/A			Individual	Individual	No	N/A
Raney et al. (2019)	80-100	Can't tell	Can't tell	Individual	Individual	No	No
Reed et al. (2013)	80-100	Yes	Can't tell	Individual	Individual	Yes	No
Wood et al. (2013)	80-100	Yes	Can't tell	Individual	Individual	No	No
Wood & Smyth (2014)	80-100	Can't tell	Can't tell	Individual	Individual	No	No
